# 

**Published:** 1893-08

**Authors:** 


### CONTENTS.

### *EDITORIALS.*

PAGE

Pyrogen., . . . . . . . . . . . , . 401

Homœopathic Aggravation, . . . . . . . . . . 401

### *MISCELLANEOUS.*

Pyrogenum (Pus from Septic Abscess.) W. A. Yingling, M. D., . 402

Potentiation Physiologically Proven. Dr. Gustav Jaeger. Translated by Dr. Fincke, M. D., . . . . . . . . . 414

Homœopathic Aggravation. S. E. Chapman, M. D., . . . . 427

Profuse Nocturnal Enuresis, . . . . . . . . . 431

Provings and Clinical Observations with High Potencies. Malcolm Macfarlan, M. D., . . . . . . . . . . 432

The International Hahnemannian Association, . . . . 443

The Pathogenesis of Pyrogen. W. A. Yingling, M. D., . . . 444

How to Cure Rhus Poisoning. Mr. R. F. Rabe, Jr., . . . . 445

### *BOOK NOTICES.*

A Practical Treatise on Materia Medica and Therapeutics. By John V. Shoemaker, A. M., M. D., . . . . . . . . 445

Diseases of the Nose and Throat. By Horace F. Ivins, M. D., . . 446

Ringworm. By J. Compton Burnett, M. D., . . . . . . 446

Stearns' Dose Book, . . . . . . . . . . 447

### *NOTES AND NOTICES.*

Orificial Surgery.—Dr. J.W. Thomson.—Dr. Charles P. Beaman.—New York State Homœopathic Hospital at Middletown.—New York Homœopathic Medical College.—Homœopathic Medical Society of Pennsylvania, . . . . . . . 447

